# A review of fermented milks: potential beneficial effects on human nutrition and health

**DOI:** 10.4314/ahs.v23i4.54

**Published:** 2023-12

**Authors:** Oktay Yerlikaya

**Affiliations:** Ege University, Faculty of Agriculture, Department of Dairy Technology, 35100, Bornova-Izmir, Turkey

**Keywords:** fermented milk, probiotics, kefir, dairy products, nutrition, human health

## Abstract

Fermented dairy products are formed during the acidification of milk through fermentation by suitable microorganisms; it contains different microorganisms in sufficient numbers and in an active state. A wide range of fermented milk products are produced and consumed around the world, including yogurt, kefir, koumiss, and yogurt beverages. There are various health benefits associated with the consumption of fermented dairy. Many studies reported that some fermented milk products have antimicrobial, antimutagenic, anticarcinogenic, and antihypertensive properties as well as provide benefits on mineral metabolism, reduce lactose intolerance symptoms and cholesterol levels. In addition to these effects, it has many other beneficial effects such as positive effects on type 2 diabetes and hypertension, antimutagen and antioxidant effects, and reduction of allergic symptoms. Dairy products including fermented milk are known to be the main carrier of probiotic microorganisms, and many clinical studies show the effects of probiotic strains on health. In this study, the effects of fermented milks on human nutrition and health are mentioned.

## Introduction

Fermented dairy products are products are characterized by different structure obtained through the fermentation of lactose in milk by different microorganisms, especially lactic acid bacteria (LAB). Various fermented dairy products with that look alike are known by different names around the world. These products form an important part of the daily diet in most countries (Yerlikaya, 2014; Ghosh et al., 2019). LAB is used as starter culture in the production of fermented milk products, including yogurt, yogurt beverage, Ayran, kefir, kumiss, sour cream, and cheese. LAB has functional and technological features that are beneficial for the production, such as acidification, proteolysis, and aroma formation. Lactobacilli, including *Lactobacillus acidophilus, Lacticaseibacillus casei, Lactobacillus gasseri, Lacticaseibacillus paracasei, Limosilactobacillus* reuteri, and *Lacticaseibacillus rhamnosus*, and *bifidobacteria* such as *Bifidobacterium animalis* subsp. *lactis, Bifidobacterium bifidum, Bifidobacterium* breve, *Bifidobacterium longum* are a major part of the probiotic LAB (Bintsis, 2018). These probiotic bacteria adapt to the acidic conditions of the stomach and a suitable environment can be created for their growth in the small intestine. Today, fermented milk products are produced both traditionally and commercially around the world (Yerlikaya, 2014).

### Popular fermented milks

Many fermented dairy products are produced and consumed all over the world, some of which show more popularity than the others. Although some of these products are produced by traditional methods, most of them are similarly commercially important. The products that are mentioned in the following section are the most consumed one around the world;

### Yogurt

Yogurt is a durable fermented milk product that can be easily produced around the world. Similarly, to milk, it also has all the essential nutritional properties. Yogurt, depending on the type of milk used during its creation as well as the ingredients added to the composition is richer than milk regarding its protein, fat, and mineral content. Due to the metabolites released during the fermentation process, its physiological and health-related properties are higher than those of the milk (Tamime & Robinson, 2009). Depending on the activity and density of the yogurt cultures used, the nutrients can be digested more easily as they are broken down at certain levels. Vitamins and active substances, such as niacin, folic acid, and choline are specifically released by the associated bacteria. *Lactobacillus delbrueckii* subsp. *bulgaricus* strains in yogurt culture metabolize lactose D (‒) and *S. thermophilus* strains to L (+) lactic acid depending on applied the strain. When these two bacteria coexist, the formulation of 47%–75% L (+) lactic acid was observed in yogurt. Higher physiological importance and absorption power are attributed to L (+) lactic acid as well as it is frequently used in infant feeding (Bostan et al., 2017). The most important feature of traditional yogurt products is the occasional formulation of a layer of cream on their surface. In traditional yogurt production, sheep or buffalo milk with higher dry matter is preferred. However, their insufficient production of milk requires the use of cow milk in the production of yogurt today (Dimitrellou et al., 2019).

### Ayran – Yogurt drink

One of the most important forms of consumption in association with yogurt is ayran or yogurt drinks, taking an important place in human nutrition. Ayran is a fermented milk beverage derived from yogurt, which is obtained by diluting yogurt or fermenting certain diluted milk as well as subsequently mixing until salt becomes homogeneous after its addition (Koksoy & Kilic, 2003). Although ayran is a drink mostly associated with the Turkish, it is produced and marketed in the form of a yogurt drink in many countries. Buttermilk, which is an important milk product which has been processed in Central Asia and Anatolia for many years and has an important place especially in the catering of the Turkish society due to its superior nutritional value, therapeutic and antimicrobial properties, ease of digestion. Moreover, ayran, which has a refreshing effect and therefore consumed with pleasure, has become a national drink in the Turkish society. Ayran has an important position in milk technology in association with the evaluation of milk, which has lost its natural qualities in a short time (Kumar et al., 2015).

### Kefir

Kefir was first produced on the skirts of the Elbrus Mountain ridge in the Caucasus as a result of the fermentation of milk with kefir grains. Kefir grains are also called as “cornflowers” and are characterized with a size ranging from 1–2 mm to 3–6 mm as well as a shape resembling a mini cauliflower. Kefir grains contain polysaccharides, a small amount of oil, and casein. Microorganisms are live in the grain in the form of symbiosis (Sarkar, 2007; De Oliveira Leite et al., 2013). During the addition of these grains to the milk, the microorganisms are introduced to the milk environment and fermentation is performed (Farnworth & Mainville, 2008). Studies reported the presence of various microorganisms, including *Levilactobacillus brevis, Lactobacillus acidophilus, Lacticasseibacillus casei, Lactobacillus helveticus, Lactobacillus lactis, Lactobacillus bulgaricus, Lactobacillus cellobiosus, Lentilactobacillus kefiri, Lentilactobacillus parakefiri, Lactobacillus kefiranofaciens* subsp. *kefirgranum, Lactobacillus kefiranofaciens* subsp. *kefiranofaciens, Lactococcus lactis, Streptococcus thermophilus, Leuconostoc mesenteroides, Leuconostoc cremoris, Bifidobacterium bifidum, Kluyveromyces* spp., *Candida* spp., *Torulopsis* spp., and *Saccharomyces* spp. (Kok-Tas et al., 2012; Prado et al., 2015).

Since kefir produced using grain is not of standard quality and difficulties arise in association with finding kefir grain, industrial kefir production has started to replace traditional production. In industrial kefir production, standardized pasteurized milk is infused with a kefir starter culture (lyophilized) and subsequently left to fermentation. Since microorganisms in the kefir culture are stable, standard quality products can be obtained (Tomar et al., 2019). Besides, the produced kefirs are offered to consumers after the addition of various fruit pulp (Kazakos et al., 2016). Ziarno et al. (2021) investigated the effect of various milk protein powder preparations on kefir on the digestibility of milk proteins in kefir. It was stated that the digestibility of milk proteins tested using the in vitro enzymatic digestion method, the addition of milk protein and the type of starter culture used showed the effect on the protein quality of kefirs.

### Koumiss

Koumiss (or kimiz) is a very old Turkish drink; it is obtained by acid and alcohol fermentation from mare's milk and propyl alcohol, butyl alcohol, propionic acid, pruvates, aldehydes, glycerine, acetone, and diacetyl, which contributes to the flavour due to the lactic acid and alcohol fermentation. Compounds, such as various ethers and volatile acids are formed. The characteristics of the koumiss vary depending on the microorganisms in the used starter culture. The presence of koumiss starter culture bacteria, including *Lactobacillus bulgaricus, Lacticaseibacillus casei, Lactococcus lactis, Lactobacillus acidophilus, Pichia* spp., *Rhodotorula* spp., *Torula lactis*, and *Mycoderma* ssp. as well as yeast, including *Kluyveromyces marxianus* and *Kluyveromyces marxianus* var. *bulgaricus* were reported by various studies (Bayat, 2020).

### Other probiotic fermented milk

The increasing consumption trend towards fermented products has been accelerated by the realization of the health benefits of such products. On the other hand, the importance of fermented milk products in human nutrition has been known for many years. The only thing that has increased in recent years is the awareness on this issue. There are about 400 types of traditional fermented milk products produced in the world. Many of these are traditionally produced locally, while others are produced on a large scale industrially. If we list the common ones among various fermented milk products and the regions where they are produced intensively; yoghurt, strained yoghurt, fruit yoghurt, ayran (Central Asia, Germany), Kefir (Central Asia, Caucasus, Czech Republic), Kumyz (Central Asia, Caucasus), Shubat (Central Asia, Caucasus, Arabia), Katik Qatyg (Central Asia, Caucasus), Quark (Germany and Northern Europe), Acidophilus milk (North America and Europe), Lebben (Arabia and Egypt), Ymer (Denmark), Boruga (Dominican Republic), Viili and Sour milk (Finland) , Calpis and Yakult (Japan), Lassi (Pakistan), Dahi (Pakistan), Matsoni (Georgia), Amasi (South Africa), Piimä (Finland), also known as Kefir.

Probiotics are living microbial microorganisms that positively affect the natural intestinal microbiota, providing a positive influence on human health. Today, several probiotic dairy products are produced using starter culture containing single or different combinations of probiotic microorganisms (Mani-López et al., 2014). Among these microorganisms, *Lactobacillus acidophilus, Bifidobacterium animalis* subsp. *lactis, Lacticaseibacillus casei, Lacticaseibacillus rhamnosus, Enterococcus faecium*, and *Saccharomyces boulardii* are the most notable (Yerlikaya, 2014). These microorganisms are also combined with yogurt bacteria, probiotic yogurt as well as yogurt beverages produced and marketed under different names. Taking probiotic bacteria into the body through fermented milk products is important for the human intestinal microbiota to reach a balance. The therapeutic effect of probiotic bacteria in dairy products depends on the survival of these bacteria during the shelf life of the product influenced by environmental conditions. The development and survival of probiotics during their passage through the stomach and intestinal system are affected by properties, such as high acidity, bile salts, the chemical composition of food carriers, and the redox potential (Ranadheera et al., 2012). Besides, the functional and technological properties of the same probiotic species also differ based on the various food ingredients and types of food-related environment (Terpou et al., 2019).

### Nutritional value and functional properties of fermented milks and Kefir

There are many health benefits associated with the consumption of fermented dairy products. It has been shown in many studies that some fermented milk products have antimicrobial, antimutagenic, anticarcinogenic, antihypertension properties and have benefits on mineral metabolism, reduce food allergy symptoms and LDL cholesterol level. It is known that dairy products are the main carriers of probiotic microorganisms, and there are many clinical studies showing the effects of probiotic strains on health (Granato et al., 2010).

The nutritional value of fermented milks depends on the type of milk used, its nutrient content and the production technology of fermented milk. The composition, nutritional value, and taste of kefir and other fermented milk products vary depending on the characteristics of the production, the type and the composition of the used milk, the characteristics of the kefir grain, or the utilization of the starter culture. Since fat, lactose, protein, mineral substances, and vitamins are contained in the milk, it is considered to be a food with high nutritional value (Arslan, 2014; Rosa et al., 2017). It was reported by researchers that CO_2_ formed in kefir facilitates digestion, bacteria that take part in fermentation synthesize group B vitamins, especially B_12_, as well as more than 90 % of the formed milk acid is L (+) milk acid which can be digested easily. Also, due to the effect of microorganisms during fermentation, changes in lactose and proteins facilitate the digestion of kefir and fermented milk, thus providing the absorption of the nutrients by the body (De Oliveira Leite et al., 2013; Prado et al., 2015). Kefir contains calcium and magnesium among minerals necessary for the nervous system, as well as it is a good source of phosphorus. The positive effects on human health of kefir are known in addition to the shown functional properties due to its many different components besides its nutritional value (Otles & Cagindi, 2013). These features are briefly mentioned in the following section.

### Antimicrobial properties

The role of microorganisms living in fermented dairy products has gained considerable attention in recent years, both for consumers and producers. Lactic acid bacteria (LAB) are the main microorganisms in milk fermentation. LAB increases the acidity of the environment by converting lactose, which is milk sugar, into lactic acid, thus creating conditions that do not allow the development of microorganisms other than LAB. LAB can also synthesize some antimicrobial compounds such as bacteriocin, reuterin and diacetyl.

The antibacterial effect of fermented dairy products results from antimicrobial compounds produced by the starter culture. While a better bactericidal effect against gram-positive microorganisms is attributed to kefir, it has a higher bacteriostatic effect against gram-negative microorganisms. Moreover, is stated that kefir has an antagonistic effect against *Escherichia coli, Listeria monocytogenes, Yersinia enterocolitica, Listeria innocua*, and *Salmonella enteriditis* (Kim et al., 2016). Furthermore, Santos et al. (2003) stated that *Lactobacilli* isolated from kefir grains show antagonistic effect against *Escherichia coli, Listeria monocytogenes, Staphylococcus typhimurium, Salmonella enteriditis, Shigella flexneri*, and *Yersinia enterocolitica* strains. The antibacterial activity of kefir against various pathogens consists of a specific group of antibodies and organic acids resulting from lactic and acetic acid produced during fermentation by acetic acid bacteria and yeasts or hydrogen peroxide produced by LAB (Kim et al., 2016; Dimidi et al., 2019).

### Gastrointestinal cell regeneration

Fermented milk products could replenish normal lactic intestinal flora by suppressing unwanted microorganisms. This antibacterial activity is also dependent on temperature, storage time, and the level of initial contamination. Kefir is an auxiliary product in patients with gastrointestinal diseases and after surgical operations (Prado et al., 2015; Ghosh et al., 2019). Murashova et al. (1997) found that a rapid decrease in *Salmonella* and *Shigella* rates was observed in children with gastrointestinal disease consuming bifidokefir (kefir containing *Bifidobacterium bifidum*) within 7–11 days. Bifidokefir containing 5×107 *Bifidobacterium* / mL was reported to have a positive effect on the intestinal flora. Besides, the appropriate ratio of *Bifidobacterium* spp. and *Lactobacillus* spp. is can be effective in the inhibition of pathogens and other bacteria.

### Cholesterol-lowering properties

The assumption that lactic acid bacteria can directly cause cholesterol level reduction in fermented milk products or living organisms has been made based on numerous *in vivo* and *in vitro* studies showing that some lactic acid bacteria lower blood cholesterol levels in the serum or model culture medium of experimental animals or human volunteers (Ziarno, 2020). High lactic acid bacteria population in kefir and the ability to bind cholesterol by 33.9% contributes to a decrease in cholesterol in the intestines. The fermentation of milk with kefir culture at 24°C for 24 hours was observed to result in the assimilation of 28%–65% of cholesterol (Bourrie et al., 2016). Yeasts, including *Saccharomyces cerevisiae*, where the invertase enzyme is insufficient were reported to have a high cholesterol effect. Wojtowski et al. (2003) noted that kefir made from sheep milk is healthier than that made from cow and goat milk due to linoleic and α-linoleic acids. Hydroxymethyl-glutaric and/or orotic acids were found to inhibit the enzyme activity involved in cholesterol synthesis. The ability of kefir to bind the loss of orotic acid, causing the accumulation of fat in the liver during fermentation of the hypocholesterolemic effect on humans, reduces the cholesterol in the intestine. The fermentation of milk with a kefir starter culture at 24°C for 24 hours was observed to contribute to the assimilation of 28%–65% of cholesterol. Yeasts, such as *Saccharomyces cerevisiae*, where the invertase enzyme is inadequate were reported to have a high cholesterol effect (Sarkar, 2007; Ahmed et al., 2013).

### Anticarcinogenic effect

The observed anticarcinogenic mechanism in fermented dairy products can be explained as the means of prevention of the onset of cancer stopping the starting tumor by cutting the working speed of the enzymes that trigger the carcinogenic effects, converting them into carcinogenic substances or making the immune system work (Gonzalez et al. 2018). Studies have shown that *Streptococcus, Lactobacillus, Leuconostoc*, and *Lactococcus lactis* subsp. *cremoris*, which are isolated from kefir, show the ability of binding mutagens. Polysaccharides in kefir grain are more effective in stopping tumor development than normal water-soluble polysaccharides. It is believed that the anticarcinogenic feature of kefir is due to polysaccharides or microorganisms produced during fermentation, especially *Lactobacillus* species (Davoodi et al., 2013; Bourrie et al., 2016; Sharif et al., 2017; Jeyaraman et al., 2019). Güzel-Seydim et al. (2003) stated that milk protein and especially high concentrations of sulfur-containing amino acids are important in the prevention of cancer.

### Organic acid production

LAB is a popular set of bacteria in association with the production of organic acids, including lactic acid, acetic acid, formic acid, succinic acid, and citric acids. LAB includes G (+) microorganisms that produce lactic acid as a major fermentation product (Nuryana et al., 2019). Lactic acid has two different isomers, namely D (−) and L (+), the amount of which in kefir depends on the yeast and bacterial microflora. The predominance of mesophilic, homofermentative lactic streptococci in the microflora was observed to result in the formation of L (+) lactic acid in an approximately 10% higher quantity in the fallopian than that in case of the D (−) lactic acid (Bintsis, 2018). Lin et al. (1999) stated that *Kluyveromyces marxianus* produces L (+) lactic acid while *Leuconostoc mesenteroides* produces D (−) lactiacid. L (+) lactic acid is physiologically important in humans and animals. In contrast, D (−) lactic acid has physiologically negative effects on the cell metabolism. The fact that bifidobacteria and most probiotic bacteria in fermented milk products produce L (+) lactic acid in 90% of the cases also emphasizes the benefits of bifidokefir consumption for children. Moreover, bifidokefir also reduces the risk of acidosis (an increase of acidic substances in the blood) compared to normal kefir (Sarkar, 2007; Yerlikaya, 2014).

### Lactose intolerance symptoms

Live microflora in fermented dairy products have different lactose fermentation abilities. For this reason, various fermented milk products may contain different amounts of lactose. The low amount of lactose in fermented milk products and the presence of β-galactosidase make kefir and fermented milk suitable for consumption for people with lactose intolerance (Suri et al., 2019). Kefir grains have a β-galactosidase effect. It can be said that kefir is as safe as yogurt in regard of lactose intolerance. Kefir contains less lactose than milk and compared to milk, it reduces the gas formed in the stomach by 54%–71% (Hertzler & Clancy, 2003). There is a need to substantiate health claims regarding foods with reduced lactose content and that reduce gastrointestinal upset caused by lactose ingestion in lactose-intolerant individuals.

### Effects on the human immune system

One of the most important chemical changes during fermentation is the proteolysis of the milk casein. The resulting peptide fractions were reported to possibly have growth-stimulating effects on kefir bacteria as well as immune system-regulating effects. After the intake of LAB in kefir, immune activities were observed in humans and various animals and lactic acid bacteria were found to increase the non-specific resistance to tumors or infections in humans or animals (Dimidi et al., 2019; García-Burgos et al., 2020).

### Bacterial colonization

The successful growth of bacteria depends on bile salt tolerances and intestinal ambient conditions (Bustos et al., 2012). 85% of Lactobacillus species isolated from kefir grains were found to be able to resist bile and most of them can attach to enterocytes. Yeasts, such as *Kluyveromyces lactis* and *Kluyveromyces ladderae* were also reported to have greater bile tolerance and be able to cling to the gut. Gastrointestinal epithelial cells contribute to important mechanisms for these organisms to settle in the intestinal tract with their ability to bind and reproduce. Retention is not necessary for the successful colonization in the gut, while clinging microorganisms create a more effective physiological effect (Rosa et al., 2017).

### Effect on type 2 diabetes

Diet and lifestyle changes can significantly reduce the risk of type-2 diabetes. People who consume low-fat dairy have a lower risk of type-2 diabetes. Although a strong inverse relationship has been reported between dairy consumption and insulin resistance among young obese adults, the relationship between milk intake and type-2 diabetes is not yet fully understood. Dairy products such as genius may show anorexic or insulinotropic effects and thus reduce the risk of diabetes (Hyon et al., 2005; Yadav et al., 2006; Nagpal et al., 2012).

Type 2 diabetes is a disorder that is regarded as the inability of the body to process and utilize blood sugar in an appropriate manner. Although the mechanism of action of dairy products in association with metabolic syndromes and type 2 diabetes is not completely known, it is thought that calcium in milk increases the burning of fat and contributes to the reduction of the inflammation pressure in the adipose tissue. Bacteria found in fermented dairy products can synthesize vitamin K, which has a positive effect against diabetes (Tong et al., 2011). Studies have reported that the consumption of fermented dairy products, such as yogurt to reduce the risk of type 2 diabetes. Besides, low-fat yogurt has been reported to be beneficial in the reduction of the risk of type 2 diabetes due to its low energy density (O'Connor et al., 2014). Moreover, diary proteins draw attention to being a suitable alternative due to their effects on postprandial blood glucose that is comparable to insulin secretagogues used in the treatment of type 2 diabetes (Patil et al., 2015). It has been argued that the water-soluble parts of kefir and kefiran also increase the uptake of glucose in skeletal muscle cells which can be potentially used in the treatment of type 2 diabetes. As a result of several prospective epidemiological studies, dairy intake was found to be associated with a lower risk of type 2 diabetes (Liu et al., 2006; O'Connor et al., 2014).

### Antihypertensive effect

Dairy products are rich in calcium, the increase of which was found to be effective for lowering high blood pressure according to a meta-analysis of randomized controlled trials (Gibson et al., 2009). That the effectiveness of potassium in milk and phosphorus in dairy products against high blood pressure were also demonstrated (Soedamah-Muthu et al., 2011). *Lactobacillus helveticus* bacteria have an inhibitory effect on hypertension and are used in the making of cheese as well as milk fermentation. In a study conducted in Finland, a milk drink was tested in mice and it was emphasized that it affected high blood pressure (Seckin and Baladura, 2011). Yildirim (2016) stated in his study on the effect of yogurt, probiotic yogurt, and kefir consumption on hypertension, that these products generally have a blood pressure-lowering effects, and that the blood pressure-lowering effect of kefir is more remarkable in normal daily use. The antihypertensive activity of the peptides is due to their ability to inactivate the angiotensin I-converting enzyme (ACE). Bioactive peptides that show antihypertensive activity are generally isolated from caseins. Casein hydrolysates were reported to produce more ACE inhibitors compared to whey protein hydrolysates, however, peptides Ala-Leu-ProMet-His-Ile-Arg (ALPMHIR) show potent antihypertensive activity and are generated during the tryptic digestion of β-lactoglobulin. Two of the sixteen proteins that are contained in kefir have been proven to possess an angiotensin-converting-enzyme-suppressing effect (Mohanty et al. 2016). Pihlanto-Leppälä et al. (2001) described an ACE inhibitory peptide in traditional fermented milk products. It is known that there are many mechanisms responsible for the effect of live microflora in fermented milk on the antihypertensive effect. It is also clear that not all fermented milk products will show similar effects.

### Antimutagen and antioxidant effect

Antioxidants have a positive effect on health as they prevent the damages resulting from free radicals, tumor and cancer development, low-density lipoproteins, as well as the oxidation of lipoproteins. The properties of antioxidative peptides are thought to be due to the chelation of metal ions, the removal of free radicals, the quenching of single oxygen atoms, and the inhibition of enzymatic and non-enzymatic lipid peroxidation (El Fattah et al., 2018). Liu et al. (2002) conducted a study on the antimutagenic and antioxidant properties of kefir and soy kefir and stated that their observed ability to show antimutagenic activity. The antioxidant properties of kefir, including its antimutagenic activity, 1,1-diphenyl-2-picrylhydrazyl (DPPH) radical scavenging activity, lipid peroxidation inhibitory activity, iron ion chelating ability, reductive effect, and antioxidative enzyme activity were evaluated by a *Salmonella* mutagenicity assay. Probiotic microorganisms were found to increase the bioavailability of nutrients by transforming the chemical components of the raw material during food fermentation.

This transformation produces antioxidant compounds in the food content (Tamang et al., 2016). Lactoferrin obtained from casein provides an antioxidative activity by inhibiting enzymatic and non-enzymatic lipid peroxidation. Whey-derived peptides help reduce the risk of many diseases, including cancer and atherosclerosis by supporting antioxidant functions. Moreover, they are rich in cysteine and glutamate, which reduce oxidative stress (Möller et al., 2008). Many mechanisms play a role in the antimutagenic and antioxidant effects of the beneficial microbiota contained in fermented milk products. Among these reasons, the reason, type of milk to be fermented, composition properties and type of fermented milk may be important.

### Prevention of allergic diseases

Many mechanisms of action of living microflora in fermented milk on the human immune system are known. Today, there is a day-by-day increase of allergic diseases. Lactic acid bacteria and probiotics, which are also used in the production of fermented dairy products, are not only useful in treatment, but also in the prevention of various allergic symptoms and signs. It has been observed in human and animal studies that the occurrence of atopic dermatitis is suppressed with the use of probiotics. The efficacy of probiotics in allergic diseases varies according to the type and dose of the selected probiotics (Yesilova et al., 2010). In addition, several studies support that probiotics may be useful in the treatment and prevention of atopic dermatitis (Uysal & Uzuner, 2012). Sjogren et al. (2009) found that high levels of *Bifidobacterium* spp. and *Lactobacillus spp. (Lactobacillus acidophilus, Lactobacillus delbrueckii*, and *Lactobacillus helveticus)* in the intestinal flora resulted in a lower incidence of allergic diseases later in life. The *Lactobacillus kefiranofaciens* M1 strain isolated from kefir grains was also reported to have an anti-allergic effect. During the maturation of fermented dairy products, the digestion of caseins was shown to facilitate the reduction of allergic reactivity, thereby increasing the level of tolerance (Tomar et al., 2017).

### Bone and dental health

In the protection of bone and dental health, the composition of the raw material from which fermented milk products are produced is also affected by the effect of bioavailability of minerals, product pH and the presence of sufficient protein. In addition, many mechanisms are known regarding the effect of living microflora in milk on the bioavailability of minerals. Bone and teeth are similar in structure. In this context, compounds such as calcium, phosphorus and protein naturally found in milk and dairy products are important for bone and dental health. Calcium is an essential component of bone, and since most of the calcium in the body is found in bone, it is reasonable to assume that bone health is closely related to calcium intake (Prentice, 2014).

Compounds in milk and dairy products can reduce the acidity that occurs after eating sugary foods. (Nagpal et al., 2012). Milk and dairy products are also sources of potassium and magnesium, which have important effects on bone health. Increasing the daily intake of calcium and protein with dairy products is very effective in improving and maintaining bone health and protecting against fractures in childhood, adolescence and later (Rizzoli, 2014).

## Conclusions

The increasing demand and therapeutic properties of a variety of highly nutritious food products contributed to the production of various dairy products by the application of cultures. Despite its vitamin, protein, and mineral content as well as its therapeutic effects, antibacterial spectrum, the widespread appearance in the gastrointestinal tract, hypocholesterolemic and anticarcinogenic effect, L (+) lactic acid content, and β-galactosidase enzyme activity, the immune system was not found to be strengthened by the bacterial colonization in association with fermented milk products. Furthermore, beneficial influences were observed due to its positive effect on type 2 diabetes, hypertension, allergic symptoms, as well as its antimutagenic and antioxidant effects. Although the beneficial effects of fermented milk products vary depending on the type and composition of milk used in its production, it also changes according to the microorganisms in the microbiota of the fermented milk type produced. Considering that fermented milk products rich in microorganism diversity such as kefir have many positive effects on human health, more research is needed on the subject. Based on these positive features, the world fermented milk industry should focus on the production of different and new fermented milk products.

## Data Availability

Not Applicable for that section.

## Code availability

Not Applicable for that section.
